# Gene essentiality, conservation index and co-evolution of genes in cyanobacteria

**DOI:** 10.1371/journal.pone.0178565

**Published:** 2017-06-08

**Authors:** Gopi Siva Sai Tiruveedula, Pramod P. Wangikar

**Affiliations:** 1 Department of Chemical Engineering, National Institute of Technology Karnataka, Surathkal, Mangalore, India; 2 Department of Chemical Engineering, Indian Institute of Technology Bombay, Powai, Mumbai, India; 3 DBT-Pan IIT Center for Bioenergy, Indian Institute of Technology Bombay, Powai, Mumbai, India; 4 Wadhwani Research Center for Bioengineering, Indian Institute of Technology Bombay, Powai, Mumbai, India; National Center for Biotechnology Information, UNITED STATES

## Abstract

Cyanobacteria, a group of photosynthetic prokaryotes, dominate the earth with ~ 10^15^ g wet biomass. Despite diversity in habitats and an ancient origin, cyanobacterial phylum has retained a significant core genome. Cyanobacteria are being explored for direct conversion of solar energy and carbon dioxide into biofuels. For this, efficient cyanobacterial strains will need to be designed via metabolic engineering. This will require identification of target knockouts to channelize the flow of carbon toward the product of interest while minimizing deletions of essential genes. We propose “Gene Conservation Index” (GCI) as a quick measure to predict gene essentiality in cyanobacteria. GCI is based on phylogenetic profile of a gene constructed with a reduced dataset of cyanobacterial genomes. GCI is the percentage of organism clusters in which the query gene is present in the reduced dataset. Of the 750 genes deemed to be essential in the experimental study on *S*. *elongatus* PCC 7942, we found 494 to be conserved across the phylum which largely comprise of the essential metabolic pathways. On the contrary, the conserved but non-essential genes broadly comprise of genes required under stress conditions. Exceptions to this rule include genes such as the glycogen synthesis and degradation enzymes, deoxyribose-phosphate aldolase (DERA), glucose-6-phosphate 1-dehydrogenase (*zwf*) and fructose-1,6-bisphosphatase class1, which are conserved but non-essential. While the essential genes are to be avoided during gene knockout studies as potentially lethal deletions, the non-essential but conserved set of genes could be interesting targets for metabolic engineering. Further, we identify clusters of co-evolving genes (CCG), which provide insights that may be useful in annotation. Principal component analysis (PCA) plots of the CCGs are demonstrated as data visualization tools that are complementary to the conventional heatmaps. Our dataset consists of phylogenetic profiles for 23,643 non-redundant cyanobacterial genes. We believe that the data and the analysis presented here will be a great resource to the scientific community interested in cyanobacteria.

## Introduction

Cyanobacteria, a group of prokaryotes, are well known for their ability to carry out oxygenic photosynthesis. They survive in different niche environmental conditions ranging from seawater to deserts and greatly contribute to the global primary production [1, 2]. This ability to sequester atmospheric carbon dioxide and photosynthetically convert it to biomass makes cyanobacteria leading candidates in biofuel research. The nitrogen fixing variety of some cyanobacteria contribute significantly to the nitrogen cycle [3] and are attracting attention as biofertilizers and for nitrogenase-dependent hydrogen production [4]. Thus, the cyanobacterial phylum shows significant diversity both in terms of their metabolic capability and habitats [5–7]. Despite this diversity, the phylum has retained a significant core genome [7, 8].

Unlike the eukaryotic algae, cyanobacteria do not produce storage compounds that are commercially attractive. Therefore, there is significant interest in metabolic engineering of cyanobacteria to produce useful products such as ethanol, butanol, butanediol, etc [9, 10]. Classical metabolic engineering involves knocking out or downregulating pathways that drain the carbon away from the product of interest [11]. Knowledge of essential genes allows mapping of critical points in metabolic networks and design of mutants with minimal wasteful experimental screening [12]. Essential genes also play a role in drug designing [13], in identifying potential targets for antibiotics in pathogenic microorganism [14] and in minimal genome construction [15]. Experimental as well as computational methods have been reported for identification of essential genes. Conventional experimental methods involve gene knockouts [16] and RNA interference [17] where viability of the organism is checked by deleting or silencing the gene. A recently developed method, random bar code transposon-site sequencing (RB-TnSeq), parallelizes this process dramatically thereby permitting genome-wide gene essentiality testing [18]. In parallel, a number of computational methods have been proposed in the past decade or so that may be time efficient. These are based on network topology [19], gene expression data [20], metabolic modelling [21], flux balance analysis (FBA) [22, 23] and ^13^C metabolic flux analysis (^13^C-MFA) [24]. FBA based methods systematically assesses the growth rate of all single gene deletion mutants. A gene is termed essential if it’s deletion adversely affects the growth rate. Likewise, synthetic lethal genes can be identified by testing viability of two or more knockouts at a time [22].

A number of studies have analyzed the core genome of photosynthetic prokaryotes. In an early study, Raymond et al. [25] performed comparative genomic analysis on different groups of photosynthetic prokaryotes for common gene orthologs to propose photosynthetic evolution. Subsequently, Shi and Falkowski [7] enumerated 682 core genes based on conservation in 13 cyanobacterial genomes. Larsson et al. [26] predicted gene orthologs with a larger genome dataset of 58 cyanobacterial genomes and identified 404 such orthologs. Beck et al. [8] categorized the clusters of likely ortholog genes (CLOGs) identified from 16 cyanobacterial strains into core, shared and unique clusters. Besides these computational studies across the cyanobacterial phylum, Rubin et al. [18] experimentally identified a total of 718 essential genes in *S*. *elongatus* PCC 7942.

Current methods of prediction of gene essentiality have certain limitations. For example, the metabolic modelling based methods are applicable only for the metabolic genes. The computational approaches used for predicting gene essentiality based on orthologs do not include all the cyanobacterial genera. In experimental methods, the experimental techniques become arduous, especially while considering multiple growth conditions. Further, it is difficult to simulate all experimental conditions that cyanobacteria may encounter in nature during evolution. This is true especially for real life stress conditions and selection pressures which decide gene essentiality. In this study, we present gene conservation index as a quick method to assess gene essentiality. Our dataset comprises of phylogenetic profiles for 23,643 non-redundant genes from cyanobacterial species of diverse genera. Apart from insights on gene essentiality, the data provides useful clues on co-evolution of genes that may be helpful in gene annotation.

## Materials and methods

### Phylogenetic profile construction

Phylogenetic profiles were constructed as described by Pellegrini et al. [27], with minor modifications. Briefly, a total of 120 completely sequenced cyanobacterial genomes were used to create a local database (S1 Table). Of these, non-redundant protein sequences from 20 genetically diverse cyanobacterial genomes were used as query sequences (S2 Table). Protein sequences from each organism were subjected to CD-HIT program to remove redundancy at 90% identity level [28]. A given query protein sequence was searched against each of the 120 cyanobacterial genomes using NCBI local BLASTP. A hit was accepted if all three of the following conditions were satisfied: (i) BLAST E-value of < 10^−5^, (ii) query coverage of > 60% and (iii) score density of > 0.6 over the aligned region (Memon et al. [29]). These conditions were imposed to minimize spurious hits that result from partial domain matches. All proteins that satisfy these criteria are listed as hits and omitted from being considered as query sequences subsequently. A “1” in the profile indicates the presence of at least one hit in the genome while a “0” indicates the absence.

### Clustering of genes and organisms

**Fig 1:**
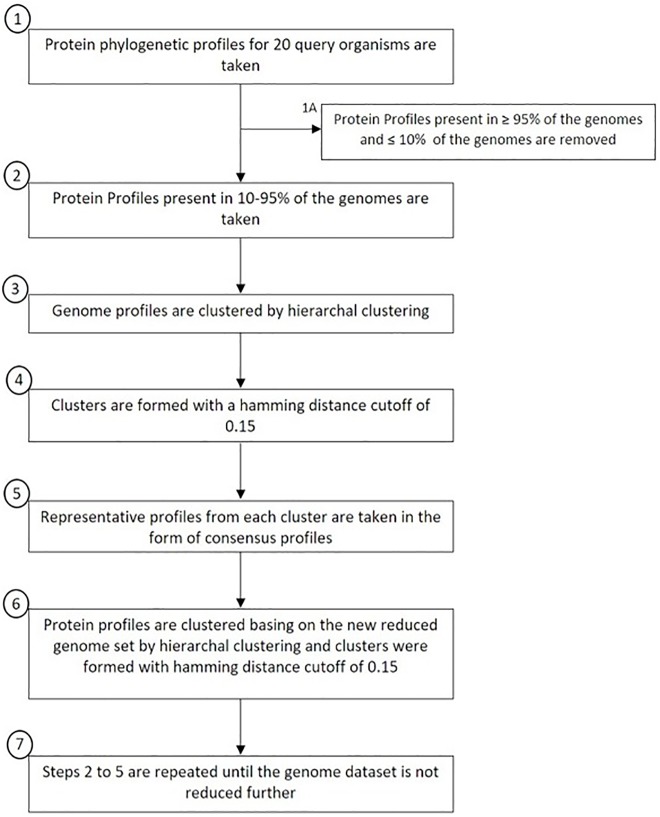
Data reduction and construction of Clusters of Co-evolving Genes (CCG). Diagrammatic representation for clustering of organisms and proteins. The method is used to reduce the genome dataset and to cluster proteins of the semi-conserved category to obtain CCG.

Genes were clustered via hierarchical clustering of their profiles with Hamming distance as the metric, a cut-off of 0.15 and average linkage. Likewise, cyanobacteria were clustered by using their profiles and a similar clustering strategy and cut-off. The merged profiles are then represented as consensus of the members of the cluster. The detailed method is depicted in Fig 1 which is used to obtain a reduced genome database.

### Modified Hamming distance

To test if any of the profile pairs are mutually exclusive, we modified the conventional Hamming distance by ignoring the elements of profiles where both the proteins are absent. The modified formula of Hamming distance is:

$$d=\frac{\sum P_{1}+\sum P_{2}-2\sum P_{1}P_{2}}{\sum P_{1}+\sum P_{2}-\sum P_{1}P_{2}}$$

where,

d = modified hamming distance,
P_1_ = Profile of gene 1, and
P_2_ = Profile of gene 2.

## Results and discussion

### Phylogenetic profile construction

Over twenty-three thousand phylogenetic profiles were constructed by using non-redundant protein coding genes from cyanobacteria as query. The protein sequences were searched against 120 cyanobacterial genomes of diverse genera using BLASTP. The database of phylogenetic profiles created not only contains the query IDs but also the hit IDs and therefore can be searched by UniProt IDs / PATRIC IDs of proteins in any of the 120 microorganisms (S3 Table). While creating the profiles, query cyanobacteria were added sequentially with the result that only a small fraction of proteins result in profiles for organisms added in later stages (S2 Table). Since only 20 cyanobacteria species were used for profile creation, some of the protein coding genes from the other cyanobacteria may not be found in our database but that number is expected to be small. Also, the genes missing in our profiles are expected to be unique genes present only in a limited number of cyanobacteria.

### Data reduction

**Fig 2:**
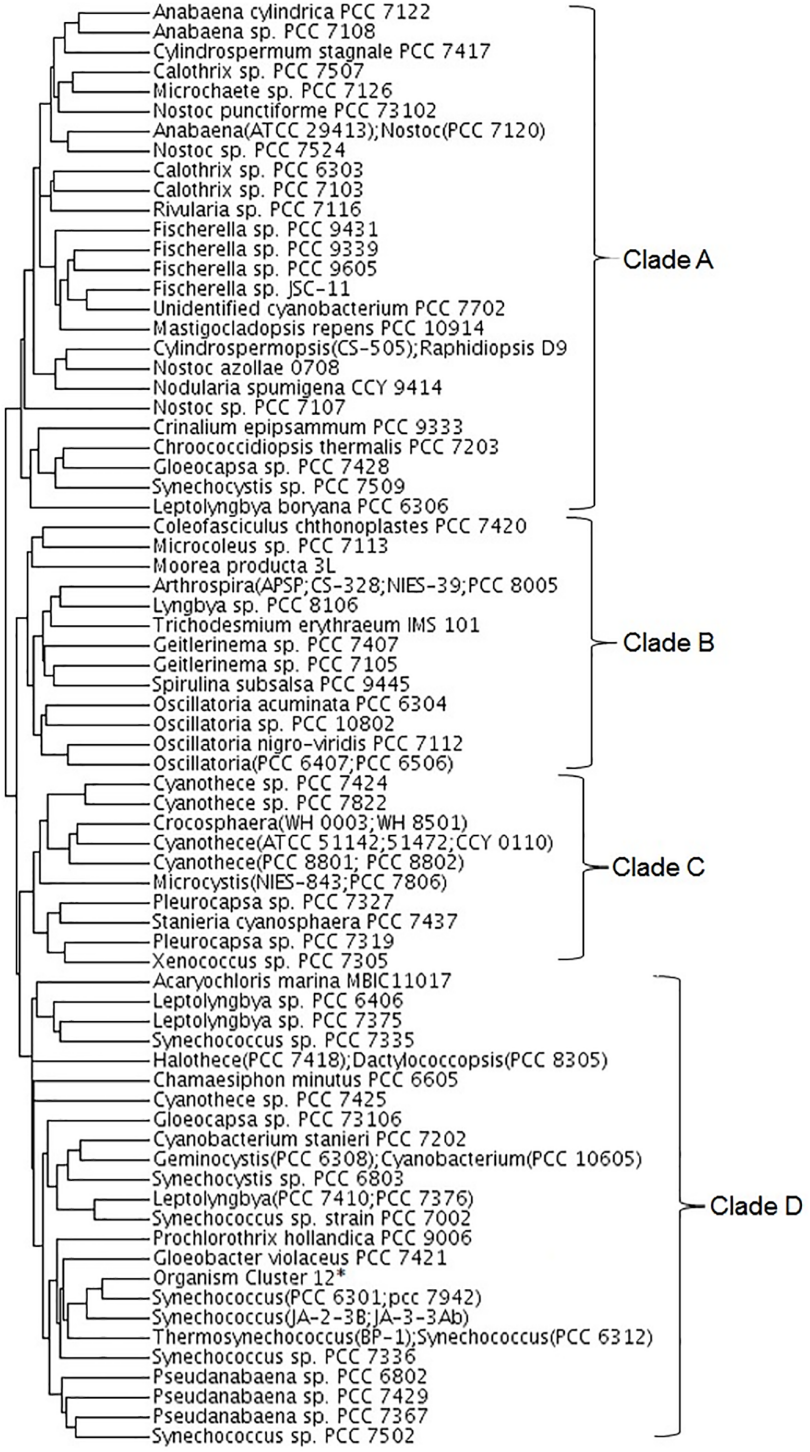
Phylogenetic tree of the 73 cyanobacterial genome groups based on clustering of their phylogenetic profiles. Profiles of 8280 semi-conserved genes were used while clustering the organisms. List of the cyanobacterial strains are given in S2 Table. * represents the organism cluster 12, whose constituents are given in S2 Table.

String length (number of genomes in the dataset) of the phylogenetic profiles was 120 based on the number of genomes used. However, some of the genera such as *Prochlorococcus* are overrepresented in the database. This may bias some of the data analysis and in turn the conclusions. Therefore, to remove redundancy, we clustered the cyanobacterial strains by using profiles of genes that are conserved in ≥ 10% but < 95% of the cyanobacterial genomes resulting in 73 clusters (Fig 1 and see methods for details). This was done to avoid the influence of either unique genes or highly conserved genes on the clustering process. Indeed, the 31 strains of *Prochlorococcus* and *Synechococcus* form a single cluster (S4 Table). Additionally, smaller clusters of 2–4 strains are formed. Further, the tree of organisms based on phylogenetic profiles agrees well with that based on alignment of concatenated protein sequences [5]. The tree can be broken down into 4 clades (Fig 2) for further clade-wise analysis as discussed in subsequent sections. Abridged profiles were then created that have string length of 73, corresponding to the 73 organism clusters, compared to the original string length of 120 (S5 Table). Conservation indices for the protein profiles were computed based on the abridged strings.

### Redundancy of profiles

**Fig 3:**
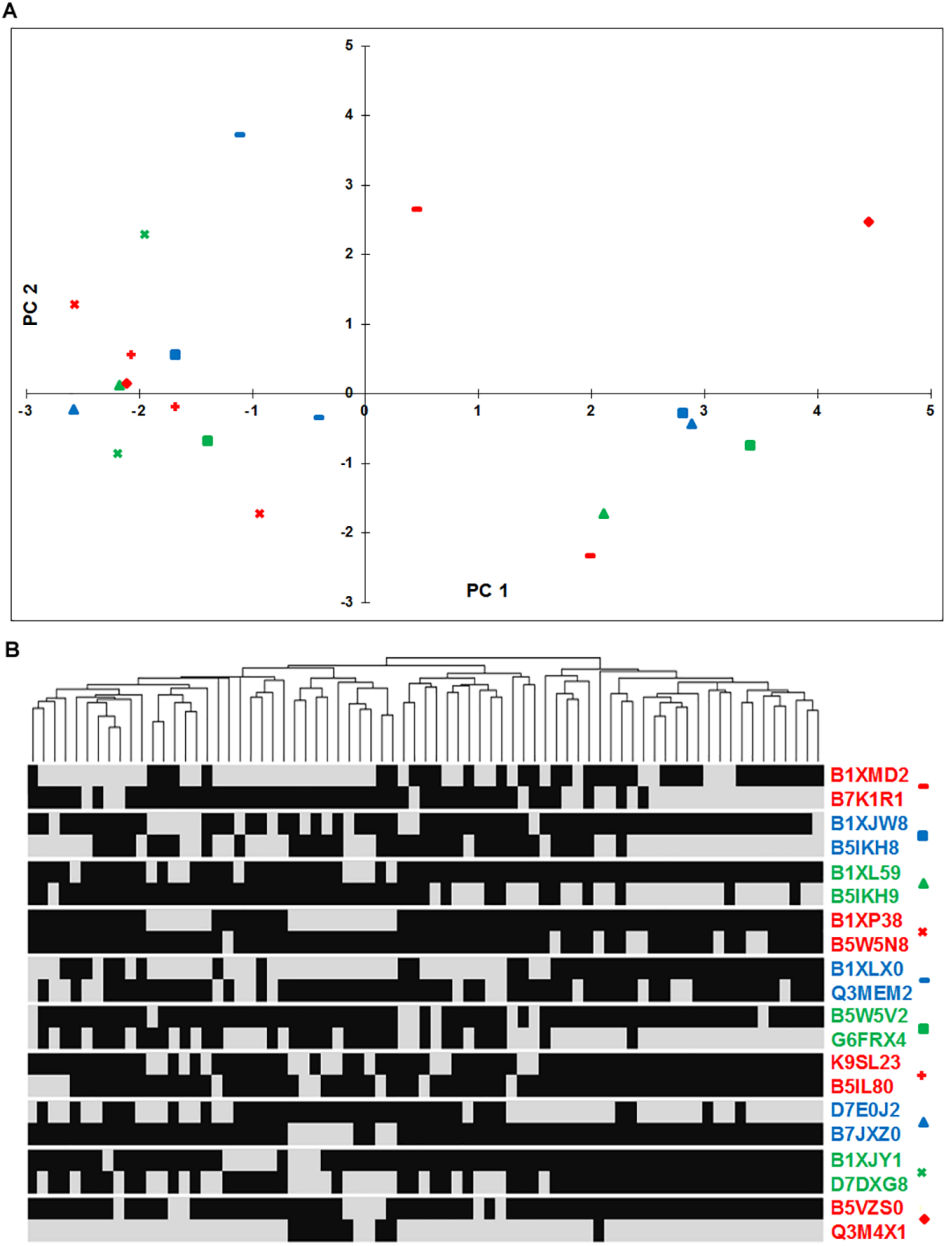
Potential redundancy in the phylogenetic profiles. Ten representative pairs of redundant profiles that show BLASTP evalue ≤ 10^−10^ but fail to meet the criteria of score density and query coverage. The redundant gene pairs are color-coded and assigned symbols in the two plots. (A) A PCA plot that shows the 10 pairs on the first two principal components and (B) Heatmap showing mutually exclusive nature of profile pairs. In the heatmap, grey and black colors indicate presence and absence of the gene, respectively.

It was of interest to check for potential redundancy in the query protein sequences used in construction of profiles. To that end, we performed an all-against-all BLASTP search of the query sequences used in profiles formation and find that 1904 of the ~ 10^5^ pairs show E-value of ≤ 10^−10^. This accounts for ~ 8% redundancy in our profiles. These potentially redundant sequence pairs formed separate profiles in our dataset as they did not satisfy the criteria of query coverage and score density. BLASTP hits may also result from partial domain matches that cannot be considered as a hit. Another potential reason for this to happen is that such protein pairs may actually be evolutionarily related with common function but may have undergone substantial evolutionary divergence. To test this hypothesis, we checked if (i) the profiles are mutually exclusive with high Hamming distance and (ii) the divergence of the two proteins correlates with the evolution of the organisms. To test mutual exclusivity, we computed modified Hamming distance of such pairs of profiles by ignoring the genomes where both genes are absent. This analysis shows that indeed a large fraction of the potentially redundant pairs are mutually exclusive. This can be visualized on the principle component analysis (PCA) plot (Fig 3A) where the pairs in question lie on opposite sides of the plot (Details regarding the proteins used in the PCA plot and heatmap are given in S6 Table). Further, a heatmap of the profiles shows that divergence of the two functionally similar proteins largely correlates with the evolution of the organisms (Fig 3B). To exemplify, profiles with query UniProt Ids B1XMD2 and B7K1R1 are mutually exclusive. Both the proteins are involved in the catalysis of the second and third steps (cysteine ligation, (EC:6.3.2.5), and decarboxylation, (EC:4.1.1.36)) in the biosynthesis of coenzyme A (CoA) from pantothenate [30] (Strauss et al, 2001). Each query protein is present in evolutionarily related subset of organisms suggesting that the divergence of the protein sequences correlates with the evolution of the organism. Likewise profiles with query UniProt IDs B5VZS0 and Q3M4X1 both represent the *phzF* (phenazine biosynthesis) family, which is part of the seven-gene operon, responsible for the synthesis of phenazine-1-carboxylic acid [31, 32]. This suggests that the potentially redundant profiles represent genes that have diverged significantly in the subsets of organisms and are not merely paralogs.

### Conservation categories

**Fig 4:**
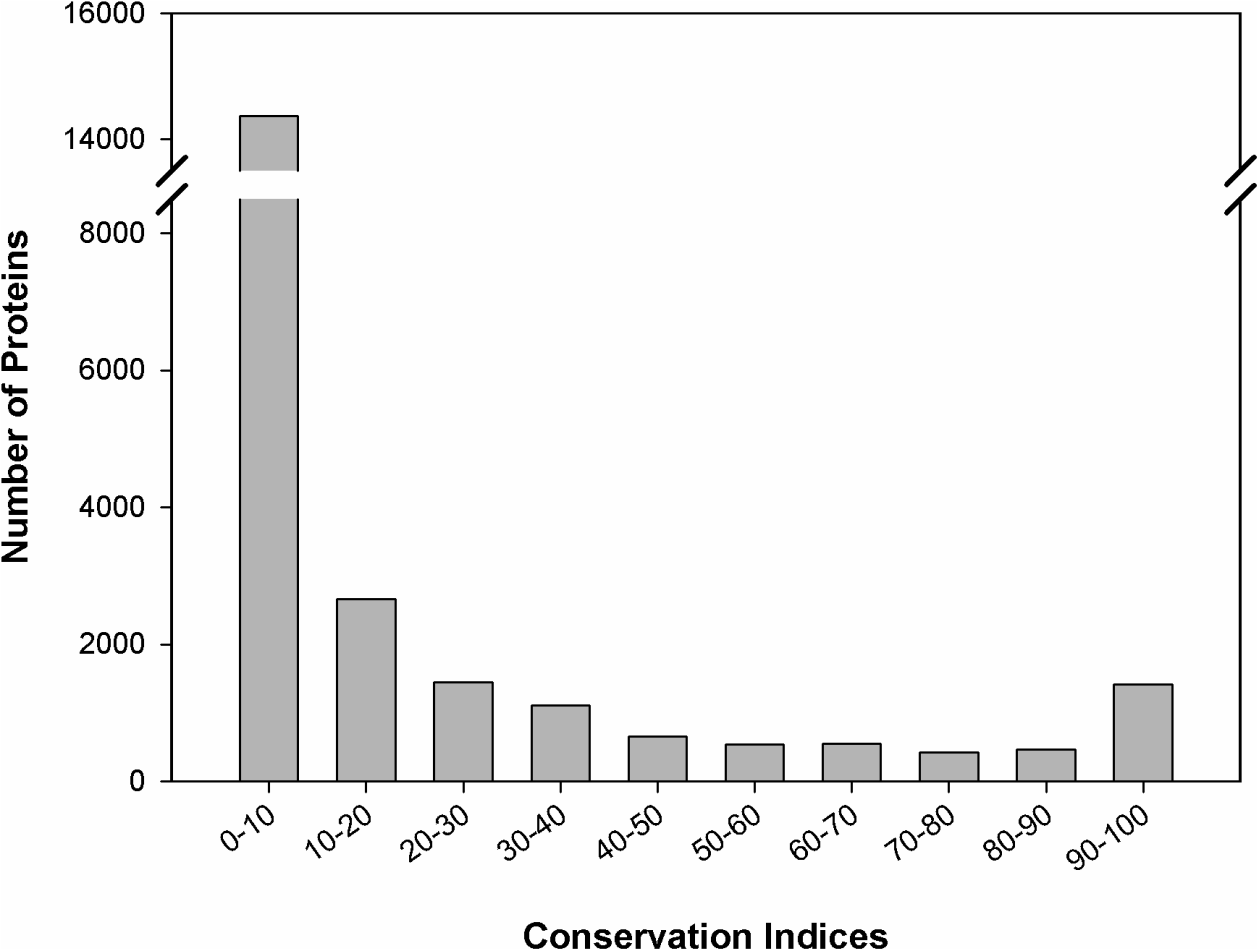
Histogram of gene conservation index (GCI) for the 23,633 phylogenetic profiles. The GCI was calculated based on presence and absence of the query gene in the 73 organism clusters of cyanobacteria.

We estimated the gene conservation index (GCI) for each of the phylogenetic profiles as the percentage of organism clusters in which the query gene is present in the reduced dataset (Fig 4). We broadly categorize all proteins of cyanobacteria as:

1. Conserved; with a GCI score of ≥ 95%,
2. Semi-conserved; with a GCI score of ≥ 10% but < 95% and
3. Unique; with a GCI score of < 10%.

About 4% of all query genes (993 profiles) are conserved across the cyanobacterial phylum (Table 1 and S7 Table). This is apart from the rRNA and tRNA genes which are also conserved but not considered here as our dataset contains only protein coding genes. In this set, around 35% of the genes are metabolic genes. The set comprises of upto 50% of the total proteins in smaller genomes such as those of *Prochlorococcus* sp. A large fraction of proteins in this category are annotated. These proteins participate in key cellular functions such as carbohydrate metabolism, energy metabolism, lipid metabolism, nucleotide metabolism, amino acid metabolism, glycan biosynthesis, metabolism of cofactors and vitamins and genetic information processing like transcription, translation and replication. Shi and Falkowski [7] have called this set the stable core.

**Table 1:** List of representatives of the conserved genes in our analysis.

UniProt IDs of Query Proteins	Protein Name	Gene Name	GO (Biological Process)	Pathway
B1XP79	Aconitate hydratase B	acnB SYNPCC7002_A1683	tricarboxylic acid cycle [GO:0006099]	Carbohydrate metabolism; tricarboxylic acid cycle.
D7DZP2	Cytochrome c oxidase, subunit I	Aazo_2640	aerobic respiration [GO:0009060]; electron transport chain [GO:0022900]; oxidative phosphorylation [GO:0006119]	Energy metabolism; oxidative phosphorylation.
Q93UM1	Enoyl-[acyl-carrier-protein] reductase [NADH]	envM SYNPCC7002_A1676	fatty acid biosynthetic process [GO:0006633]	Lipid metabolism; fatty acid biosynthesis.
B1XN91	GDP-L-fucose synthase	fcl SYNPCC7002_A2832	'de novo' GDP-L-fucose biosynthetic process [GO:0042351]	Nucleotide-sugar biosynthesis; GDP-L-fucose biosynthesis via de novo pathway.
B1XLQ9	2-isopropylmalate synthase	leuA SYNPCC7002_A1356	leucine biosynthetic process [GO:0009098]	Amino-acid biosynthesis; L-leucine biosynthesis.
B1XQ71	1,4-alpha-glucan branching enzyme	glgB SYNPCC7002_A1865	glycogen biosynthetic process [GO:0005978]	Glycan biosynthesis; glycogen biosynthesis.
B1XNE1	Biotin synthase	bioB SYNPCC7002_A0309	biotin biosynthetic process [GO:0009102]	Cofactor biosynthesis; biotin biosynthesis.
B1XJ16	30S ribosomal protein S1	rpsA SYNPCC7002_A0955	translation [GO:0006412]	-
B1XPW9	Arsenical resistance operon repressor, ArsR family	SYNPCC7002_A0590	transcription, DNA-templated [GO:0006351]	-
B1XNN3	DNA polymerase III, delta subunit	holA SYNPCC7002_A1567	DNA replication [GO:0006260]	-

About 35% of all the query genes (8,280 profiles) are semi-conserved. These include genes involved in niche functions such as nitrogen fixation, defense response to viruses, response to stress conditions, etc. Although only semi-conserved at the level of cyanobacterial phylum, some of these genes are conserved in their respective clades and this needs to be examined further.

About 61% of the query genes (14,370 profiles) are unique and present only in < 10% of the organism clusters. A large fraction of these proteins are not annotated. It is likely that these proteins are conserved only at species level performing a highly niche function.

We have specifically analyzed for the circadian clock genes which are involved in the regulation of global gene expression patterns, interactions with the genes of key metabolic pathways, the timing of cell division and in chromosome compaction [33, 34]. We checked for the conservation index of these genes and we find that the oscillator genes *kaiB* and *kaiC* fall under the conserved category while *kaiA* belongs to the semi-conserved category. Among the genes that provide inputs to the circadian clock, the gene *cikA* is semi-conserved while the gene *ldpA* is conserved. Among the genes that transmit output of the clock to downstream genes, the gene *sasA* is conserved.

**Fig 5:**
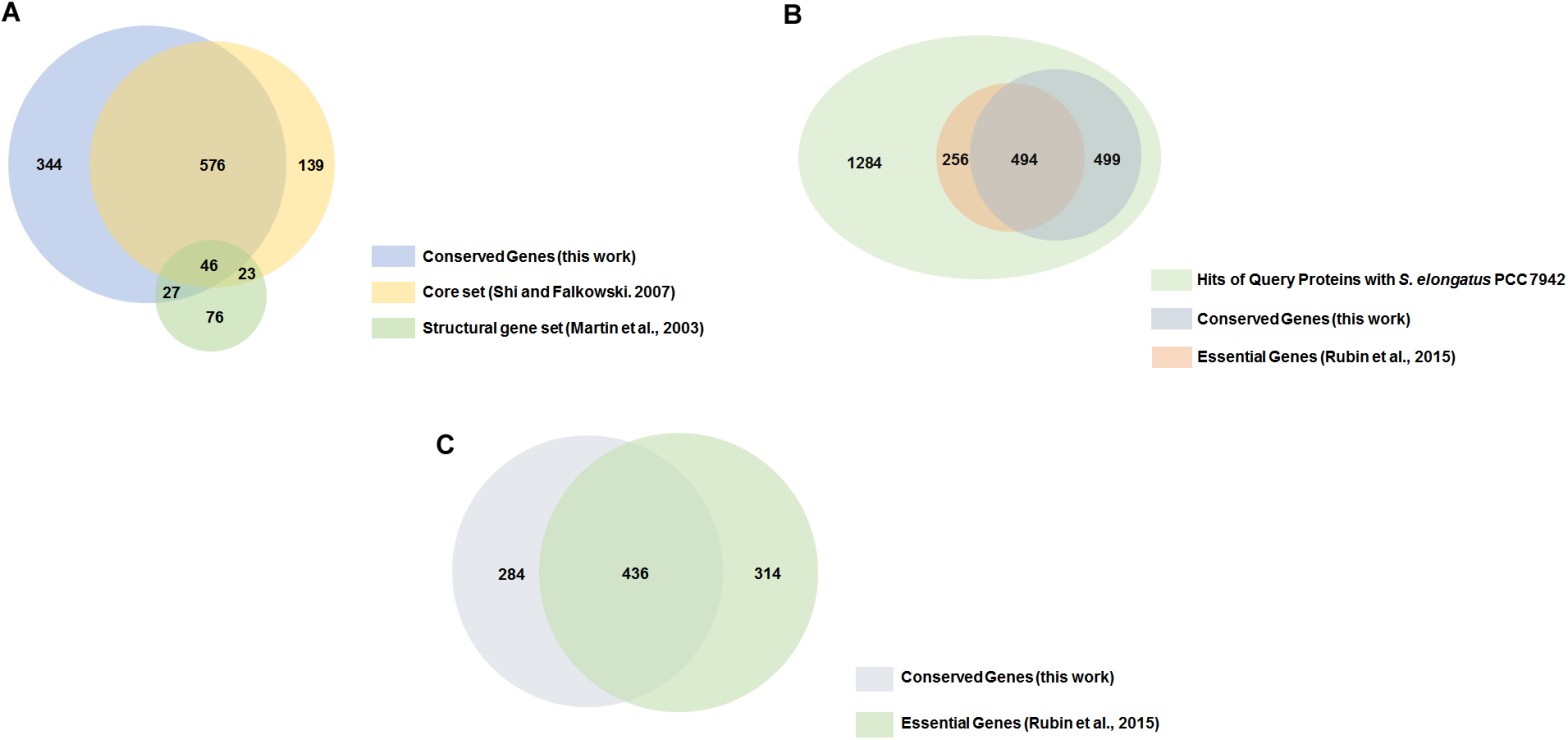
Overlap of conserved genes (this work) with core set proteins of Shi and Falkowski [7], structural genes of Martin et al. [35] and essential genes of Rubin et al. [18] shown for *S*. *elongatus* PCC 7942 for which experimental data is available. (A) Overlap of conserved genes (this work), core set proteins [7] and structural genes [35]. (B) Conserved genes (this work) and essential genes [18]. (C) Conserved genes without clustering of microorganisms and removing the microorganism redundancy and the overlap with essential genes [18].

Initially Martin et al. [35] reported such a set in cyanobacteria which they called structural genes. Later Shi and Falkowski [7] reported such a gene set in cyanobacteria which they called the stable core. Only few cyanobacterial genomes were available at that time. Our analysis benefits by the availability of a much larger number of genomes. We further improve upon the results by clustering of the organisms which removes the bias introduced by the overrepresentation of genomes from certain genera. Further, the 95% cut-off used by us helps remove errors introduced due to incomplete genome sequencing. A large number of conserved genes are common between our analysis and that presented by Martin et al. [35] and Shi and Falkowski [7] (Fig 5A). However, there is some disagreement between the data sets. For example, genes *psbJ* and *psbL* (with UniProt IDs Q8RSW0 and Q8RSW1) are involved in assembly of oxygen evolving complex and unidirectional flow of electrons [36, 37] are semi-conserved in our dataset but conserved in the dataset of Shi and Falkowski [7]. Gene *psbF* (with UniProt ID Q8RSW2) is involved in the assembly of photosystem II and secondary electron transport mechanism [38] is semi-conserved in our dataset is one of the structural gene of Martin et al. [35]. These discrepancies may be due to differences in the genome datasets used in the three studies. Our analysis includes symbiotic strains which may lack some of the core metabolic genes.

### Relationship between conservation level and annotation

We found that around 89% of the conserved genes are functionally annotated (Table 2). We hypothesize that conserved genes code for core functions and may be essential. This may have attracted the attention of the scientific community toward these genes. On the other extreme, only 24% of the unique genes are functionally annotated. These proteins are typically unique to specific genera which may not have been well studied. At the intermediate level, around 47% of the semi-conserved genes are functionally annotated.

**Table 2:** The number of annotated and unannotated genes in the three conservation categories.

	Conservation Categories					
	Conserved (≥ 95%)		Semi-conserved (10–94%)		Unique (less than 10%)	
	Annotated	Unannotated	Annotated	Unannotated	Annotated	Unannotated
Number of genes	891	102	4,413	3,867	3,505	10,865

### Gene essentiality and conservation index

We wanted to test if the gene conservation index is indicative of gene essentiality. We find that there is a significant overlap between the sets of conserved genes (this work) and essential genes as determined experimentally in *S*. *elongatus* PCC 7942 by Rubin et al. [18] (S8 Table and Fig 5B). Majority of the essential genes belong to the conserved set of genes while majority of the non-essential genes belong to the semi-conserved or unique category (Table 3 and S1 Fig). The average GCI values of the essential and non-essential genes are 88% and 68% respectively. The minor discrepancy between the conservation category and essentiality may result from (i) clade specific conserved genes that may not be detected as phylum-wide conserved genes and (ii) the choice of experimental conditions may not cover the types of evolutionary pressures that organisms experience in nature. The genes *psbF* and *menF* are examples of the first case. The gene *psbF* (cytochrome b559 subunit beta) with UniProt ID Q8RSW2 is involved in the assembly of photosystem II and secondary electron transport mechanism [38]. The gene is semi-conserved in the entire phylum but is conserved in clade C. The gene *menF* (isochorismate synthase) with UniProt ID B1XJE2 is involved in the conversion of chorismate to isochorismate [39]. The gene is conserved in clades A and D but semi-conserved in the entire phylum. The genes *fbp* and *gnd* are examples of the latter category. The gene *fbp* (fructose-1,6-bisphosphatase class 1) with UniProt ID B1XNG1 is involved in reductive phase of Pentose Phosphate Pathway (PPP), and catalyses the removal of a phosphate group from fructose 1,6- bisphosphate to form fructose-6-phosphate [40]. The gene *gnd* (6-phosphogluconate dehydrogenase, decarboxylating) with UniProt ID B1XM87 is a key enzyme in PPP involved in the conversion of 6-phospho-D-gluconolactone to D-ribulose 5-phosphate [41]. The genes are conserved in our data but are non-essential in *S*. *elongatus* PCC 7942 [18]. This may be due to the fact that the PPP pathway may be used under certain stress conditions that are not accounted for in the experimental studies on gene essentiality.

**Table 3:** Essentiality of genes in each conservation category.

	Conserved	Semi-Conserved	Unique
Essential	494	242	14
Non-Essential	374	1089	88

In order to validate the reduction of the genome dataset, we compared the gene essentiality of non-reduced genome dataset (consisting of 120 genomes) with that of the reduced dataset (consisting of 73 genomes) (Fig 5C). We find that a total of 720 genes were under conserved category among which 436 genes were essential in the non-reduced genome. This number is smaller compared to the number of conserved genes (993) and essential genes (494) in the reduced dataset.

## Genes of central metabolic pathways

We analyzed the gene conservation indices and experimentally determined essentiality [18] for enzymes of select central carbon pathways (Table 4 and S9 Table). We find agreement between the two datasets. For example, on analysis of ten enzymes of the glycolytic pathway, we find that seven were conserved, six of which are essential in *S*. *elongatus* PCC 7942 [18]. Similarly, for Calvin cycle, we find that eight of the ten conserved enzymes are essential. However, we observe a disagreement with the data of Rubin et al. [18] in the cases of gene *fbp* (fructose-1,6-bisphosphatase class 1) and *gap* (glyceraldehyde-3-phosphate dehydrogenase). The disagreement between GCI and essentiality for certain enzymes may be due to the conditions opted for carrying out the experiments for essentiality in *S*. *elongatus* PCC 7942 by Rubin et al. [18].

**Table 4:** Comparison of enzymes in central metabolic pathways with the essential and conserved genes of other datasets.

Pathway	Gene Name	Protein Name	GCI^a^	Essentiality^b^	Core Set^c^	Structural Gene^d^
**Glycolysis**						
	*glk*	Glucokinase	II	NE	Y	N
	*pgi*	Glucose-6-phosphate isomerase	I	E	Y	N
	*pfkA*	6-phosphofructokinase	II	NE	N	N
	*fbaB*	fructose-bisphosphate aldolase class I	II	-	-	N
	*tpiA*	triosephosphate isomerase	I	E	Y	N
	*gap*	glyceraldehyde-3-phosphate dehydrogenase	I	NE	N	N
	*pgk*	phosphoglycerate kinase	I	E	Y	N
	*gpm*	phosphoglycerate mutase	I	E	N	N
	*eno*	enolase	I	E	Y	N
	*pyk*	pyruvate kinase	I	E	N	N
**TCA Cycle**						
	*Cs*	Citrate synthase	I	E	Y	N
	*acnB*	aconitate hydratase	I	E	N	N
	*icd*	isocitrate dehydrogenase	II	E	N	N
	*sucD*	succinyl-CoA synthetase, alpha chain	II	-	-	-
	*hdrB*	Heterodisulfide reductase, subunit B	II	NE	N	N
	*frdA*	Succinate dehydrogenase flavoprotein subunit	II	NE	N	N
	*sdhB*	Succinate dehydrogenase iron-sulfur protein subunit	II	-	-	N
	*mdh*	malate dehydrogenase	II	NE	N	N
**PS I**						
	*psaB*	photosystem I P700 chlorophyll a apoprotein A2	I	E	Y	N
	*psaC*	Photosystem I iron-sulfur center	II	E	Y	Y
	*psaD*	Photosystem I subunit II	I	E	Y	N
	*psaE*	Photosystem I reaction center subunit IV	I	NE	Y	N
	*psaF*	Photosystem I reaction center subunit III	I	NE	N	N
	*psaI*	photosystem I subunit VIII	II	Ambiguous	N	-
	*psaJ*	Photosystem I reaction center subunit IX	II	E	N	N
	*psaK*	photosystem I reaction center subunit X	II	NE	N	N
	*psaL*	Photosystem I reaction center subunit XI	I	Beneficial	N	Y
	*psaM*	Photosystem I reaction center subunit XII	II	NE	N	N
	*psaX*	photosystem I 4.8kDa protein	II	-	-	-

^a^Gene Conservation Index: I: Conserved (present in ≥ 95% of organism cluster), II: Semi-conserved (present in 10–94% of the organism cluster).

^b^Essentiality as experimentally assessed in *S*. *elongatus* PCC 7942 [18]: E: Essential, NE: Non-essential.

^c^Core Set [7]: Y: Yes (Conserved), N: No (Not Conserved).

^d^Structural Gene [35]: Y: Yes (Structural Gene), N: No (Non-structural Gene).

### Clusters of Co-evolving Genes (CCG)

**Fig 6:**
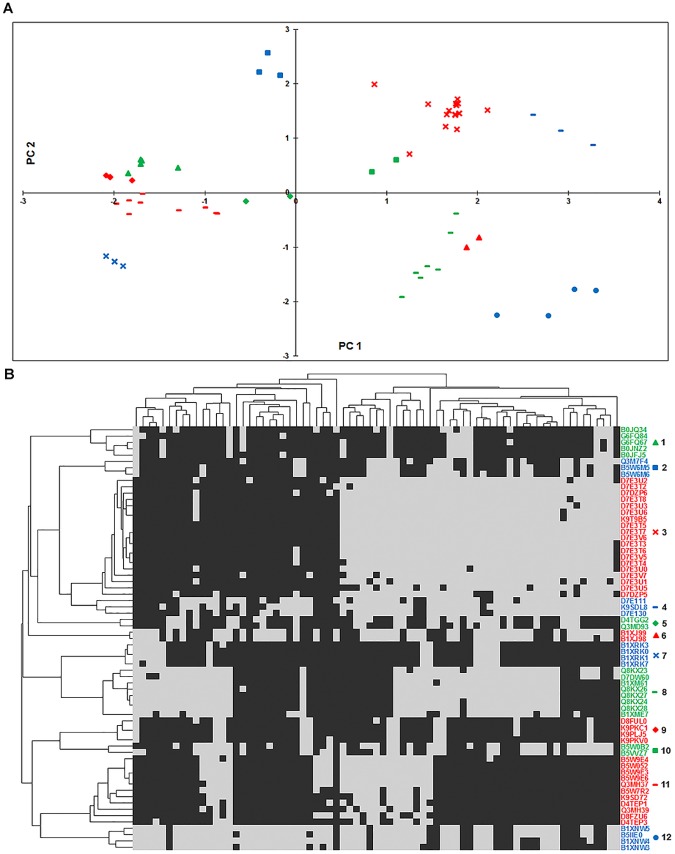
Clusters of Co-evolving Genes (CCG). Twelve representative CCGs are shown in (A) PCA plot where principle component analysis (PCA) of all the semi-conserved profiles (genes) was performed to plot only the representative genes and (B) Heatmap. Genes of a cluster are color-coded in the two plots. Phylogenetic profiles of genes are clustered using hierarchical clustering with Hamming distance as the metric, average linkage and a cutoff of 0.15. The representative phylogenetic profiles (genes) are then clustered again to depict their relatedness. Organism clusters in the heatmap are based on their genome profiles depicting their evolutionary relationships. In the heatmap, grey and black colors indicate presence and absence of the gene, respectively. Details of the clusters are as follows: Cluster 1: transposase enzymes, Cluster 2: acetamidase/formamidase enzymes, Cluster 3: nitrogen fixation genes, Cluster 4: SH3 domain protein, Cluster 5: sodium symporter proteins, Cluster 6: phosphate ABC transporter proteins, Cluster 7: ATP synthase subunit enzymes, Cluster 8: hydrogenase enzymes, Cluster 9: CRISPR associated proteins, Cluster 10: TPR repeat-containing proteins, Cluster 11: gas vesicle proteins, Cluster 12: ABC transporter proteins.

It has been well established that genes of related function experience common evolutionary pressure and hence tend to co-evolve [27]. To that end, we clustered the semi-conserved genes using Hamming distance as metric and a cutoff of 0.15. A few selected clusters (Table 5 and S10 Table) are shown in Fig 6B in the form of heatmap and all the clusters are reported in the S11 Table. The extent of co-evolution can also be visualized in the form of a principal component analysis (PCA) plot (Fig 6A). To exemplify, cluster 11 has 11 proteins, of which eight are gas vesicle proteins, two are uncharacterized (with UniProt IDs D4TEP3 and Q3MH39) and one is *ArsA* (arsenite-activated ATPase with UniProt ID B5W7R2). The gas vesicle proteins are small, hollow, gas filled protein structures found in several cyanobacteria which allow their positioning at favorable depth for growth [42, 43]. The gene *ArsA* is involved in active extrusion of heavy metals and is associated with gas vesicle biogenesis proteins [44–46]. Cluster 9 has 4 proteins, of which three are Clustered Regularly Interspaced Short Palindromic Repeats associated (CRISPR) proteins, and one is uncharacterized (with UniProt ID D8FUL0). CRISPR associated proteins provide acquired resistance against mobile genetic elements (virus, transposable elements and conjugative plasmids) [47, 48]. The uncharacterized proteins present in the above clusters may have similar functions related to the proteins present in the respective clusters and further analysis on these proteins will give additional information about their functions.

**Table 5:** Genes in the cluster one of CCG. Column A to C describes various identifiers and attributes of the genes used. Column D describes the gene conservation index.

UniProt IDs	Protein Name	Gene Name	GCI
D8FUL0	Putative uncharacterized protein	OSCI_520004	17.8
K9PKC1	CRISPR-associated helicase, Cas3 family	Cal7507_2972	15.1
K9PKV0	CRISPR-associated protein Csc3	Cal7507_2975	15.1
K9PLJ5	CRISPR-associated protein Csc1	Cal7507_2973	15.1

## Conclusion

We present systematic analysis of phylogenetic profiles of cyanobacterial genes. Clustering of the organisms helps prepare a reduced dataset of genomes, which is an important step in the entire analysis. Further, we propose gene conservation index (GCI) as a ready measure to predict gene essentiality in the cyanobacterial phylum. A large majority of the conserved genes (this study) have been found to be essential (Rubin et al, 2015). The CCG, obtained upon clustering of the semi-conserved genes, provide useful clues on the function of unannotated genes. We also present useful ways of visualization of the data in the form of heatmap and principal component analysis (PCA). We believe that the data presented here would serve as a useful resource to the scientific community.

## Supporting information

S1 Fig Histogram of gene conservation index (GCI) for the essential and non-essential genes of *S*. *elongatus* PCC 7942. Essentiality is obtained from the experimental studies of Rubin et al, 2015 [18].
(TIF)

S1 Table List of the 120 cyanobacterial genomes used in the analysis. (XLSX)

S2 Table List of query organisms used while constructing the profiles. Columns C and D describe the number of proteins used to construct the profiles and number of proteins present in the genome, respectively. The organisms are listed in sequential order in which they were used to form the profiles.
(XLSX)

S3 Table Phylogenetic profiles of 23,643 query proteins drawn from twenty genetically diverse cyanobacterial genomes. Columns A-D describe the various identifiers and attributes of the query proteins. Column E depicts the presence or absence of the query protein in the genome of while column F provides the best hit. Likewise, presence of the query proteins in 120 cyanobacterial genomes and the respective best hits are presented in columns G to IJ.
(XLSX)

S4 Table Clusters of 120 cyanobacterial strains. Column A denotes the name of the cyanobacteria and the thick borders represents a cluster. Column B describe the cluster number. The cyanobacterial strains are arranged in the order shown in Fig 2 from top to bottom.
(XLSX)

S5 Table Phylogenetic profiles of 23,643 query proteins drawn from 20 diverse cyanobacterial genomes across 73 clusters of cyanobacteria. Columns A-D describe the various identifiers and attributes of the query proteins. Columns E to BY depicts the presence or absence of the query protein in the respective cluster of organisms. Column BZ denotes the gene conservation index (GCI) for the profile. Columns CA to CD depict if the gene is conserved in clades A to D.
(XLSX)

S6 Table List of genes used for PCA plot and heatmap generation to check for mutually exclusive and diverse genes. Column A to C describes about various identifiers and attributes of the genes used. Column D describes the Gene Conservation Index (GCI). All the genes are color coded according to the Fig 3.
(XLSX)

S7 Table List of conserved genes in our analysis and their gene ontology classification. (XLSX)

S8 Table Conservation level of the genes from *Synechococcus elongatus* PCC 7942 based on the reduced dataset and compared with experimental results on gene essentiality by Rubin et al. [18] and database of core proteins by Shi and Falkowski [7]. Column A describe the Protein UniProt IDs of query genes used in the study, Column B to D describe the genes of *S*. *elongatus* PCC 7942.
(XLSX)

S9 Table Conservation level of enzymes in key metabolic pathways based on reduced dataset and compared with experimental results on gene essentiality by Rubin et al. [18], database of core proteins by Shi and Falkowski [7] and structural genes by Martin et al. [35]. (XLSX)

S10 Table List of genes used for PCA plot and heatmap generation to check the group of co-evolving genes. Column A to C describes various identifiers and attributes of the genes used. Column D describes the Gene Conservation Index (GCI). All the genes are color coded according to the Fig 6.
(XLSX)

S11 Table List of co-evolving genes. Column A describes the cluster number. Column B to D describes various identifiers and attributes of the genes used. Column E denotes the GCI.
(XLSX)
