# Supplementary figures and images for: Gene essentiality, conservation index and co-evolution of genes in cyanobacteria
Source: PLoS One. 2017 Jun 8;12(6):e0178565. doi: 10.1371/journal.pone.0178565 (PMC5464585; doi:10.1371/journal.pone.0178565)

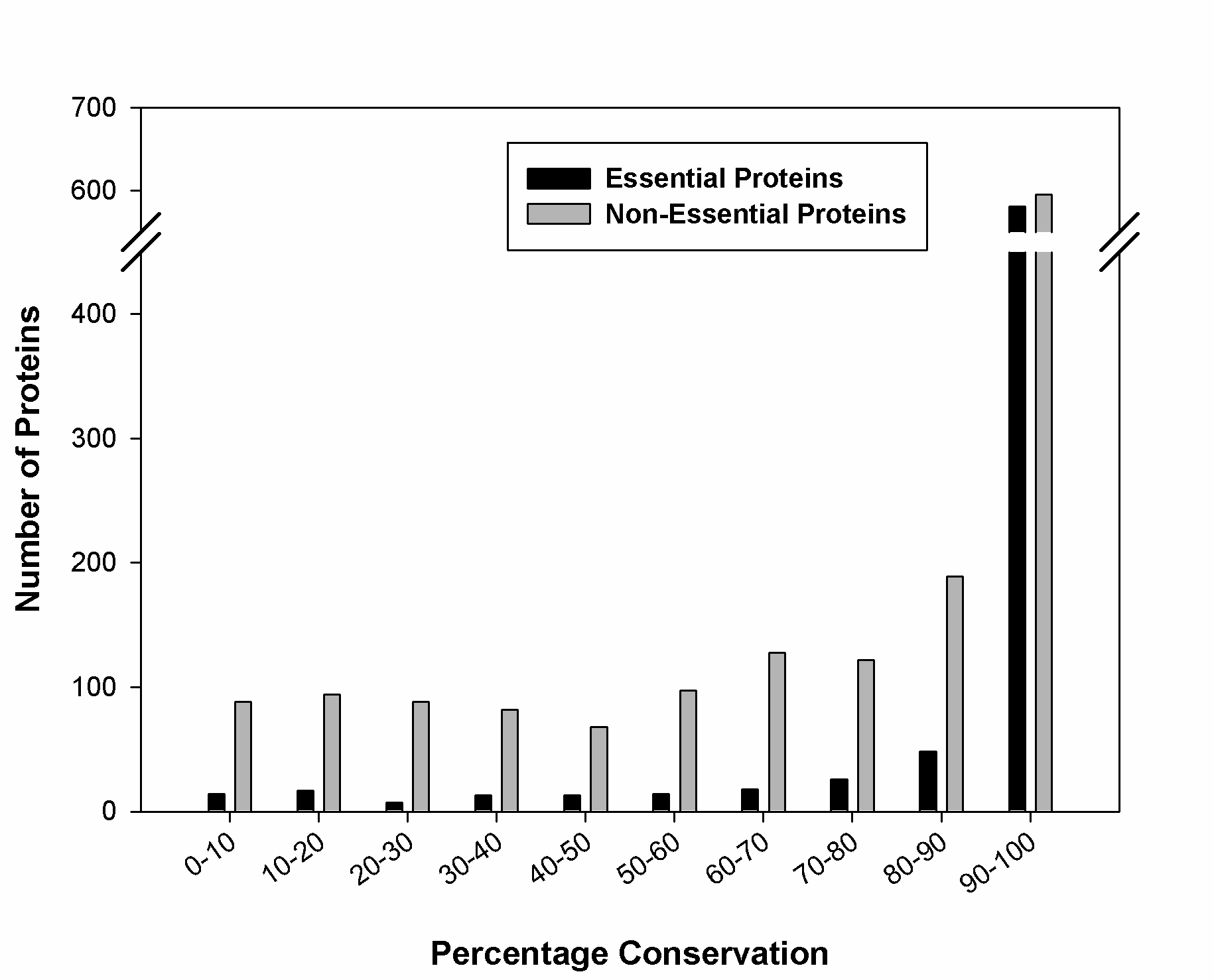

Supplement: S1 Fig — Essentiality is obtained from the experimental studies of Rubin et al, 2015 [18]. (TIF) [file pone.0178565.s001.tif]
